# Editorial on emerging neuroimaging tools for studying normal and abnormal human brain development

**DOI:** 10.3389/fnhum.2015.00127

**Published:** 2015-03-11

**Authors:** Christos Papadelis, P. Ellen Grant, Yoshio Okada, Hubert Preissl

**Affiliations:** ^1^BabyMEG/EEG facility, Fetal-Neonatal Neuroimaging and Developmental Science Center, Boston Children's HospitalBoston, MA, USA; ^2^Division of Newborn Medicine, Department of Medicine, Boston Children's Hospital, Harvard Medical SchoolBoston, MA, USA; ^3^Department of Radiology, Boston Children's Hospital, Harvard Medical SchoolBoston, MA, USA; ^4^fMEG Center, Institute for Medical Psychology and Behavioural Neurobiology, University of TuebingenTuebingen, Germany; ^5^Department of Obstetrics and Gynecology, University of Arkansas for Medical SciencesLittle Rock, AR, USA

**Keywords:** developmental disabilities, magnetoencephalography (MEG), pediatric neuroscience, pediatric neuroimaging, brain development

Research on human brain development has seen an upturn in the past few years due to increasing use of noninvasive neuroimaging tools for studying the anatomy and function of the developing brain. Here we gathered innovative studies of human brain function and development using magnetic resonance imaging (MRI), near infrared spectroscopy (NIRS) and magnetoencephalography (MEG) with experimental paradigms suitable for pediatric research. These modalities are without significant risk to the developing brain, generally require minimal patient preparation, and are well tolerated by children when performed by experienced teams. A review of recent studies of human brain development using these advanced neuroimaging tools is quite timely, since we are witnessing advances not only in the instrumentation optimized for the pediatric population, but also in research focused on the human fetuses in utero, neonates, and older children. MRI methods such volumetric T1 imaging and Diffusion Tensor Imaging (DTI) are being used more frequently in children to determine the gross anatomy and structural connectivity of the developing brain. Functional MRI and NIRS are being used to assess the hemodynamics of neurovascular responses and functional localization in development (Govindan et al., 2014; Horowitz-Kraus et al., 2014). MEG complements electroencephalography (EEG) as the only other technique capable of directly measuring the developing brain neural activity in an entirely passive manner with MEG being superior to EEG in its ability to localize activity during development. MEG and EEG can be used to assess electrophysiological functions of the developing human brain (Edgar et al., 2014). Findings from multiple neuroimaging methods can be combined to answer specific scientific questions regarding pediatric pathology (Brown et al., 2014; Hunold et al., 2014; Papadelis et al., 2014) or typical human brain development. Although MEG is still being used relatively rarely in pediatric studies, recent developments in this technology (Roberts et al., 2014) are beginning to demonstrate its utility in both the basic and clinical neuroscience of brain development (Edgar et al., 2014; Rezaie et al., 2014; Sowman et al., 2014; Taylor et al., 2014). Biomagnetic techniques also offer a direct noninvasive way to assess the functional brain and heart activity of human fetuses in utero. Unlike electric fields, magnetic fields produced by the electrical activity in the heart and brain of the fetus are not attenuated by the vernix, a waxy film covering its entire skin. Biomagnetic instruments specifically designed for fetal studies have been developed for this purpose. Fetal MEG studies using such a system have shown that both spontaneous brain activity and evoked cortical activity can be measured from outside the abdomen of pregnant mothers (Muenssinger et al., 2013). Fetal MEG and Magnetocardiography (MCG) may become clinically very useful for implementation and evaluation of intervention programs in at-risk populations. Biomagnetic instruments have also been developed for specifically measuring the brain activity in newborns, infants, and older children (Roberts et al., 2014). MEG studies have shown the usefulness of MEG for localizing active regions in the brain and also for tracking the longitudinal maturation of various sensory systems. Studies of pediatric patients are beginning to show interesting functional pathology in autism spectrum disorder (Doesburg et al., 2013; Edgar et al., 2014), cerebral palsy (Papadelis et al., 2014), epilepsy (Hunold et al., 2014; Khan et al., 2014; Tanaka and Stufflebeam, 2014), and other neurological and psychiatric disorders (Down syndrome, traumatic brain injury, Tourette syndrome, hearing deficits, childhood migraine) (Larson and Lee, 2014). The current research topic gathers studies from different research groups studying the human brain development by using advanced neuroimaging tools. Neuroimaging can offer critical information about both normal as well as abnormal human brain development. Most of the currently available tools are designed for adult use. Hardware and methods especially tailored for pediatric use remain under continual refinement. Issues relating to compliance are now mitigated by new software developments such as head motion tracking and motion correction. Such technological advancements to address specific issues germane to pediatric populations will likely promote wider adoption of neuroimaging for both clinical as well as research purposes.

## Conflict of interest statement

The authors declare that the research was conducted in the absence of any commercial or financial relationships that could be construed as a potential conflict of interest.
